# Malnutrition as predictor of survival from anti-retroviral treatment among children living with HIV/AIDS in Southwest Ethiopia: survival analysis

**DOI:** 10.1186/s12887-019-1823-x

**Published:** 2019-12-04

**Authors:** Abdu Oumer, Mina Edo Kubsa, Berhanu Abebaw Mekonnen

**Affiliations:** 1 Department of Public Health, College of Heath Sciences and Medicine, Wolkite University, Wolkite, Ethiopia; 2 Department of Nutrition and Dietetics, School of Public Heath, Bahir Dar University, Bahir Dar, Ethiopia

**Keywords:** Under nutrition, Children, HIV/AIDS, Treatment outcome, Predictor

## Abstract

**Background:**

Approximately 70% of HIV positive people live in Africa where food insecurity and under nutrition are endemic. However the impact of malnutrition on treatment outcome is not clear. This study assessed the effect of under nutrition on Anti-Retroviral Therapy treatment outcome among pediatric age group living with HIV/AIDS in Public Hospitals, Southwest Ethiopia.

**Method:**

**A** retrospective cohort study was conducted on records of 242 pediatric children in Guraghe zone Public Hospitals. Also median, mean, standard deviation and interquartile range were calculated. Life table, hazard function and survival function were plotted. Log rank test with 95% confidence interval of mean survival time was done. The nutritional status data were managed via WHO Anthros plus and BMI for age Z score was calculated. To assess effects of nutritional status on mortality, both Bivariate and multivariate cox proportional hazard regression was conducted with crude (CHR) and adjusted hazard ratio (AHR) (95% confidence interval and *p* value). *P* value of less than 0.05 was used as cut off point to declare statistical significance.

**Results:**

A total of 243 records of pediatric ART records with mean age of 11.6 (± 3.8 years) were reviewed. About 178 (73.3%) have got therapeutic feeding on the course of ART treatment. Whereas significant number of children, 163 (67.1%) reported to had eating problems. A total of 13 (5.3%) children were dead with incidence density of 11.2 deaths per 1000 person years. There is significantly higher survival time among well nourished (11.1 years with 95% CI: 10.8 to 11.4) as compared to underweight children (9.76 with 95% CI: 9.19 to 10.32 years). Underweight children had almost three fold increase incidence of death (AHR = 3.01; 95% CI: 0.80–11.4). Similarly children with anemia had higher incidence of death than children without anemia (AHR = 1.55; 95% CI: 0.49–4.84).

**Conclusions:**

Low nutritional status at the start of ART evidenced by underweight and anemia were found to be predictors of survival among HIV positive children. There should be improved, sustained and focused nutritional screening, care and treatment for children on ART follow up.

## Introduction

### Background

Nutrition is a critical component of treatment, care, and support, for chronic Human Immune Virus (HIV) care especially among children [1]. Human Immune Virus (HIV) and acquired immune deficiency syndrome (AIDS) and under nutrition interact in a vicious cycle: HIV-induced immune impairment and increased risk of infection can worsen malnutrition, lead to nutritional deficiencies through decreased food intake, malabsorption syndromes, and increased need and higher nutrient loss [2]. Children with HIV/AIDS have reduced appetite and ability to consume food, as well as a higher incidence of diarrhea resulting in malabsorption and nutrient losses [2, 3], which makes malnutrition common phenomena [4]. In addition nutritional status has an impact on the adherence of ART drug intake which is important for good treatment outcome and increased expectancy [5].

Nutritional status modulates the immunological response to HIV infection, affecting the overall clinical outcomes [6, 7]. Weight loss is an important predictor of death from AIDS. The links between nutrition and HIV/AIDS increase the negative effects of HIV infection on human development at individual, household, community and national levels [8].

Early mortality with advanced disease of HIV is common features of individuals who are already enrolled on ART drugs [9]. Those people living with HIV (PLWHA) and co-infected with tuberculosis (TB), leads to even greater metabolic stress and risk of malnutrition [10].

Approximately 36.9 million people globally live with HIV and AIDS, out of these 3.2 million were children under 15 years, including 220,000 new cases of pediatric age group. Sub Saharan Africa harbors 70% of global burden where food insecurity and under nutrition are endemic [1].

Under nutrition is a significant factor affecting human immune deficiency virus (HIV) care and treatment in resource limited settings [11]. Poor nutritional status and its intersection with food insecurity, poverty, and co-infections also pose a serious threat to efforts to combat HIV/AIDS mainly through hindering favorable treatment outcomes [5, 12]. Undernourished children with PLWHA at start ART had 2–6 times increased risks of death in the first 6 months of ART [3, 13].

In addition, untreated HIV infection increases energy needs by as much as 10% in asymptomatic adults, 20–30% in symptomatic adults, and 50–100% in children with weight loss [14]*.* Study from Northwest Ethiopia showed overall prevalence of malnutrition, underweight, stunting and wasting was 42.9, 41.7, 65 and 5.8% respectively [15].

Various level of ART treatment outcome, incidence rate of 16.9 deaths per 1000 child-years. With death rate of 4.81% [16], while another study [17] showed cumulative probabilities of survival at 3, 6, 12, and 24 months of ART were 0.96, 0.94, 0.93, and 0.92 respectively. Majority (90.2%) of the deaths occurred within the first year of treatment.

Studies has shown that baseline CD4 level, WHO staging and other parameters as important predictors of child survival from ART [15–17]. Those children whose age less than 18 months (AHR = 4.39 (1.15–17.41)), CD4 percentage < 10 (AHR = 2.98 (1.12–7.94), WHO clinical stage (III & IV) (AHR = 4.457 (1.01–19.66) were found to be significant predictors of child survival from HIV/AIDS [16]. Similarly anemia (hemoglobin level < 10 g/dl) (AHR = 2.44, 95% CI: 1.26, 4.73), increased hazards of mortality [17]. Malnourished patients were 1.5 times at risk of death compared to well-nourished group (AHR = 1.5, (0.648, 3.287), but not statistically significant [18].

Generally previous studies have been conducted in Ethiopia addressing the predictors of mortality among ART clients, yet there are a limited number of studies with this specific age group and proper assessment of nutritional status was not used. Furthermore there is no sufficient evidence in the specific area regarding the effects of malnutrition (using BMI for age for children) on Survival of children from HIV/AIDS in the study area. Thus, this particular study aimed to address the magnitude of malnutrition and more specifically its effects on the survival status of children from HIV/AIDS in this particular area which will be an important input to the area and the region Country as well.

## Methods and materials

### Study setting

Guraghe zone is one of the administrative zones in southern nations and nationalities region. It has 13 districts and two town administrations. Wolkite town is the capital of Guraghe zone which is found 425 km and 158 km from Hawassa and Addis Ababa respectively on the way to Jimma. There are five hospitals with four governmental and one private (Non-governmental) hospital. There were 758 pediatric children who were HIV positive from August 2013–September 2017 (during the past 5 years), in those public hospitals. This study was conducted from June 1 to 20/ 2018.

### Study design

A retrospective cohort study design was conducted using records from August, 2013 to September, 2017. All pediatrics patients under the age of 18 years on ART in Guraghe zone, SNNPR, Ethiopia were source population.

### Eligibility criteria

All randomly selected records of pediatrics patients (6 months – 18 years) on ART from the selected Hospitals in Guraghe zone, SNNPR, Ethiopia were study population. Records with incomplete data on nutritional status (weight, height), age, and/or ART treatment outcome were excluded from the study. Records in which the intake form, register or follow up form lacks the aforementioned variables were excluded. Cases which transferred to other facilities (transfer outs) and those cases of death with confirmed accidental death due to injury (unrelated competing causes of death) were excluded.

### Sample size determination

The sample size for the first objective was calculated based on single population proportion formula. Using incidence of mortality among ART clients (*P* = 2.3%) from study done in Zewditu hospital [19] and margin of error, 5% at 95% confidence interval as follows.

$$ n={\left( Z\alpha /2\right)}^2\ \frac{P\left(1-P\right)}{d^2} $$

$$ n={(1.96)}^2\ \frac{0.023\left(1-0.023\right)}{(0.05)^2} $$

The sample size became 35. For the second specific objective the sample size was calculated using specific software version 13.0 (Stata corps Ltd), using 80% power, 5% type I error and estimates of outcome, the sample size was 347. But as the available records of pediatric ART clients is below the calculated sample size (243), all records of eligible children were included in the study.

### Sampling procedures

Since, in Guraghe zone there are five hospitals first we stratify the hospitals and from the lists of hospitals we selected two governmental hospitals out of four which have higher case load. Using the average 5 year case load of each hospitals, we proportionally allocated the sample size to each hospitals. And records were selected using simple random sampling by computer random number generator. Then using unique ART number or medical record number (MRN) the clients’ card were retrieved. However, the total number of eligible study participants were below the calculated sample size, all records of children (243) from the selected Hospitals were included in the study.

### Data collection methods

Quantitative data were collected using structured and cross checked checklist. The check list included information on weight, socio-demographic characteristics, treatment related issues, ART treatment outcome (as death or survival from the card or register). The data were collected by trained data collectors. Data were collected by 30 health professional (BSc nurses and health officers), who have good knowledge on ART therapy. The number of data collectors assigned to each hospital was based on their previous case load. .

### Variables of the study

The dependent variable of this study is ART treatment Outcome (time to death) while age at enrollment, sex, parents survival status, nutritional status (BMI for age score), functional status, ART adherence, CD4 at the start, WHO stage at the start, cotrimoxazole prophylaxis, INH prophylaxis, co morbidities, ART regimen, eligibility criteria (CD4 or WHO staging) were independent variables.

### Operational definitions

Death: when the child had approved records of death in his medical record where it is not due to accidental unrelated causes. Censored: those records or ART clients who did not develop the outcome (death) at the ends of the study. Malnutrition is when the enrollment BMI for age z score of the child is below − 2 SD. Under nutrition for children was defined as BMI for age z- score of < − 2 SD (thinness or underweight), using World Health Organization (WHO) 2006 and 2007 reference standards. .

### Data quality control

Two days training was given to the data collectors before the actual data collection. Principal investigators and supervisors monitored and check the daily progress of the data collection. The information collected were cross checked with different sources (intake forms, ART register and ART chronic follow up form). The data were entered in to EpiData software and was restricted by legal values and other parameters to minimize errors. The data were also be entered by two independent data entry clerks and then cross checked for possible errors of data entry. Mismatched data were cross checked with the hard copy and corrected accordingly.

### Data processing and analysis

The raw data were entered in to Epi-Data software version 3.1 and exported to SPSS version 20 for analysis. Data were presented in frequency, percentages, tables, and graphs. To analyze time to death among ART clients from the day of enrolment, survival analysis was done. Also median, mean, standard deviation and interquartile range were calculated. Life table, hazard function and survival function were plotted. To compare average survival time among different characteristics, log rank test with 95% confidence interval of mean survival time and P of log rank test was done. Patients were followed retrospectively from the day of enrolment to ART till they develop the outcome or being censored.

The nutritional status data (age, weight, height) were entered in to WHO Anthros software and BMI for age Z score was calculated automatically. Then it was exported to SPSS version 20. Nutritional status was categorized in to Malnourished (BMI for Age Z score below − 2) while, the others as normal nutritional status (BMI for Age > = − 2, including obese and overweight) as over nutrition is rare among RVI clients.

To assess effects of nutritional status on mortality, both Bivariate and multivariate cox proportional hazard regression were conducted with crude (CHR) and adjusted hazard ratio (AHR) (95% confidence interval and *p* value). *P* value of less than 0.05 was used as cut off point to declare statistical significance.

### Ethical consideration

The study was approved by Wolkite University Institutional and Health Ethical Review committee. Letter of cooperation was taken from the university to the zonal health office and to the respective hospitals sequentially. Before data collection Informed consent was obtained from the respective hospital managers after full explanations of the study procedure. Then during the actual data collection all hard copy and softcopy data were under full protection in the hands of the investigators. The collected data will not be used for other purpose than the study primary objectives. Written informed consent was obtained from hospital administrator on the behalf of the ART data in their hospital. No tissue sample or human experiment was done.

## Results

### Socio demographic characteristics

A total of 243 records of pediatric ART records with mean age of 11.6 (± 3.8 years) were reviewed and included in the study. More than half children (55.1%), came from rural areas. Only about 41% of children parents live together while 10.7% and 30.5% lost their mother and father at some period% of time. About 209 (86%) of children live with their parents, while others with their relatives or lonely. Almost all, 240 (98.8%) had a care giver who can take care of them, of whom 86% of them were parents either father or mother of the child (Table 1).

**Table 1:** Socio demographic characteristics of children on ART follow up in Guraghe zone public hospitals, 2019

Characteristics	Frequency	Percentage
Sex		
Male	116	47.7
Female	127	52.3
Residence		
Rural	134	55.1
Urban	109	44.9
Parental Status		
Both alive	101	41.6
Mother Died	26	10.7
Father died	74	30.5
Both died	19	7.8
Separated/widowed	23	9.5
Child live with		
Parent	209	86.0
Sister/brother	3	1.2
Aunt/uncle	12	4.9
Grandparents	17	7
with other (non parents)	2	0.8

A total of 239 (98.4%) children got reported nutritional counseling during their ART follow up period. While about 178 (73.3%) have got therapeutic feeding on the course of ART treatment. Whereas significant number of children, 163 (67.1%) reported to had eating problems. Of whom, 156 (95.7%), 68 (41.7%) and 81 (49.7%) had loss of appetite, swallowing difficulty and vomiting respectively (Table 2).

**Table 2:** Nutrition related characteristics of ART children on ART follow up in Guraghe zone public hospitals, 2019

Variables	Frequency	Percentage
Nutritional Counseling		
No	4	1.6
Yes	239	98.4
Therapeutic feeding		
No	65	26.7
Yes	178	73.3
Eating Problem (*n* = 243)		
No	80	32.9
Yes	163	67.1
Loss of Appetite (*n* = 163)		
No	7	4.3
Yes	156	95.7
Swallowing Difficulty (*n* = 163)		
No	95	58.3
Yes	68	41.7
Vomiting (*n* = 163)		
No	81	49.7
Yes	82	50.3

A total of 191 (78.6%) children reported to have at least one opportunistic diseases. Out of which, 100 (52.4%), 21(11%) and 95 (49.7%) children had pneumonia, Tuberculosis and diarrheal disease respectively (Table 3). About 156 (64.2%) of children started ART based on the WHO criteria of being under 15 years of age, while others were admitted based on cd4 or WHO staging criteria with median age of ART start at age of 5 years. A total of 84 (34.6%) of children were put on ART immediately after HIV confirmation date while 70.2% started with in 1 month of HIV confirmation. Majority of children, 174 (71.6%) were on AZT-3TC-NVP ART regimen with mean baseline hemoglobin of 12 g/dl and CD4 count of 483 cells/mm3.

**Table 3:** ART related conditions of children on ART follow up in Guraghe zone public hospitals, 2019

Characteristics	Options	Frequency	Percentage
Presence of OIs (*n* = 243)	No	52	21.4
	Yes	191	78.6
Pneumonia (*n* = 191)	No	91	47.6
	Yes	100	52.4
Tuberculosis (*n* = 191)	No	170	89
	Yes	21	11
Diarrheal Diseases (*n* = 191)	No	96	50.3
	Yes	95	49.7
Skin infections (*n* = 191)	No	54	28.3
	Yes	137	71.7
Current Adherence (*n* = 243)	Good	202	83.1
	Fair	34	14.0
	Poor	7	2.9
INH prophylaxis (*n* = 243)	No	28	11.5
	Yes	215	88.5
Cotrimoxazole Prophylaxis (*n* = 243)	No	23	9.5
	Yes	220	90.5
Anemia (*n* = 243)	No	210	86.4
	Yes	33	13.6
Virological failure (*n* = 243)	No	218	89.7
	Yes	25	10.3
ART Regimen shift (*n* = 243)	No	230	94.7
	Yes	13	5.3

**Fig. 1:**
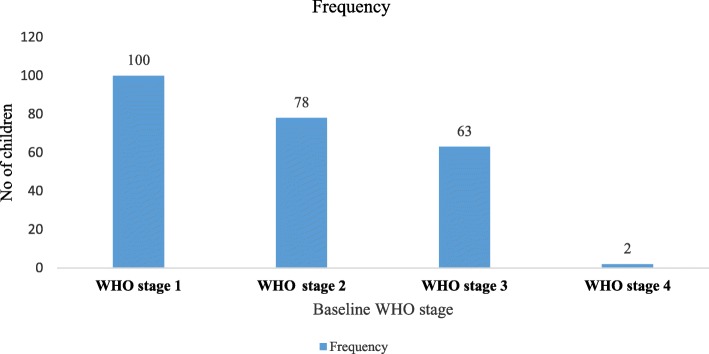
Baseline WHO stage of children on ART follow up in Guraghe zone public hospitals, 2019. (Blue refers to frequency r number of participants in each category)

A total of 41.2 and 32.2% of children on ART were on WHO stage I and II during enrollment in chronic HIV care while only 2 children were classified as stage IV. Regarding the developmental milestone of children, 109 (44.9%) of children had normal development while, 38 (15.65) had regressed developmental milestone at enrollment to ART care (Fig. 1).

**Fig. 2:**
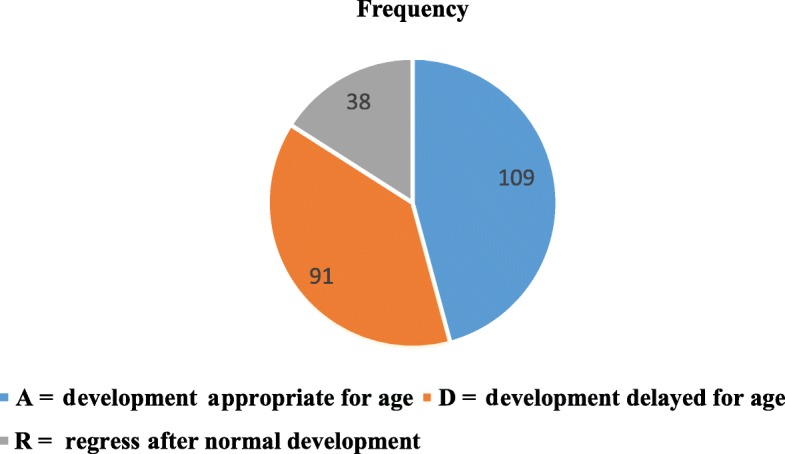
Baseline functional status of children on ART follow up in Guraghe zone public hospitals, 2019. (Blue refers to Appropriate for age; Grey refers to development delayed for age; and blue black refers to regress normal development)

Concerning the ART adherence at the time of last ART visit, 202 (83.3%) had good adherence while 2.9% had poor ART adherence which need adherence support. In addition, 88.5% and 90.5% of children had taken INH and cotrimoxazole prophylaxis respectively. A total of 33 children (13.6%) reported to have anemia and 25 (10.3%) had virological failure, persistent increase in viral count above 1000 cells/mm^3^. Accordingly, 13 (5.3%) of clients got shift of ART regimen from first line to second line, which was mainly due to clinical and immunologic failure (Table 3) (Fig. 2).

### ART treatment outcome

**Fig. 3:**
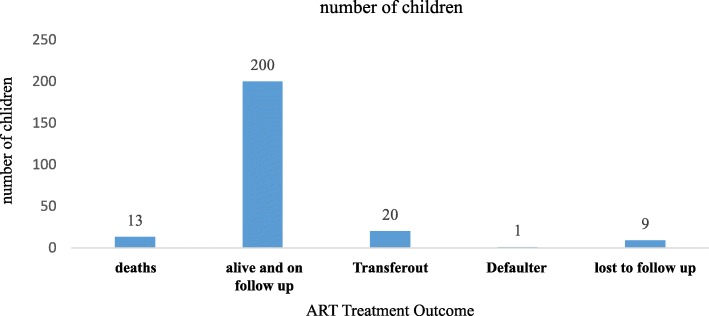
ART treatment outcomes of Children on ART follow up in Guraghe Zone public Hospitals, 2019. (Blue refers to frequency r number of participants in each category)

A total of 200 (82.3%) of children were active on ART follow up while, 13 (5.3%) were dead during the course of their chronic HIV care with incidence density of 11.2 deaths per 1000 person years of follow up (Fig. 3) (Table 4).

**Table 4:** Life table for time to death among children on chronic ART follow up in Guraghe zone public hospitals in 2019

Start Time in year	Number Entering Interval	Number Withdrawing during Interval	Number Exposed to Risk	Number of Terminal Events	Proportion Terminating	Proportion Surviving	Cumulative Proportion Surviving at End of Interval	Std. Error of Cumulative Proportion Surviving at End of Interval	Probability Density	Std. Error of Probability Density	Hazard Rate	Std. Error of Hazard Rate
0	243	7	239.5	1	.00	1.00	1.00	.00	.004	.004	.00	.00
1	235	23	223.5	11	.05	.95	.95	.01	.049	.014	.05	.02
2	201	33	184.5	0	.00	1.00	.95	.01	.000	.000	.00	.00
3	168	32	152.0	1	.01	.99	.94	.02	.006	.006	.01	.01
4	135	31	119.5	0	.00	1.00	.94	.02	.000	.000	.00	.00
5	104	33	87.5	0	.00	1.00	.94	.02	.000	.000	.00	.00
6	71	22	60.0	0	.00	1.00	.94	.02	.000	.000	.00	.00
7	49	7	45.5	0	.00	1.00	.94	.02	.000	.000	.00	.00
8	42	11	36.5	0	.00	1.00	.94	.02	.000	.000	.00	.00
9	31	12	25.0	0	.00	1.00	.94	.02	.000	.000	.00	.00
10	19	17	10.5	0	.00	1.00	.94	.02	.000	.000	.00	.00

**Fig. 4:**
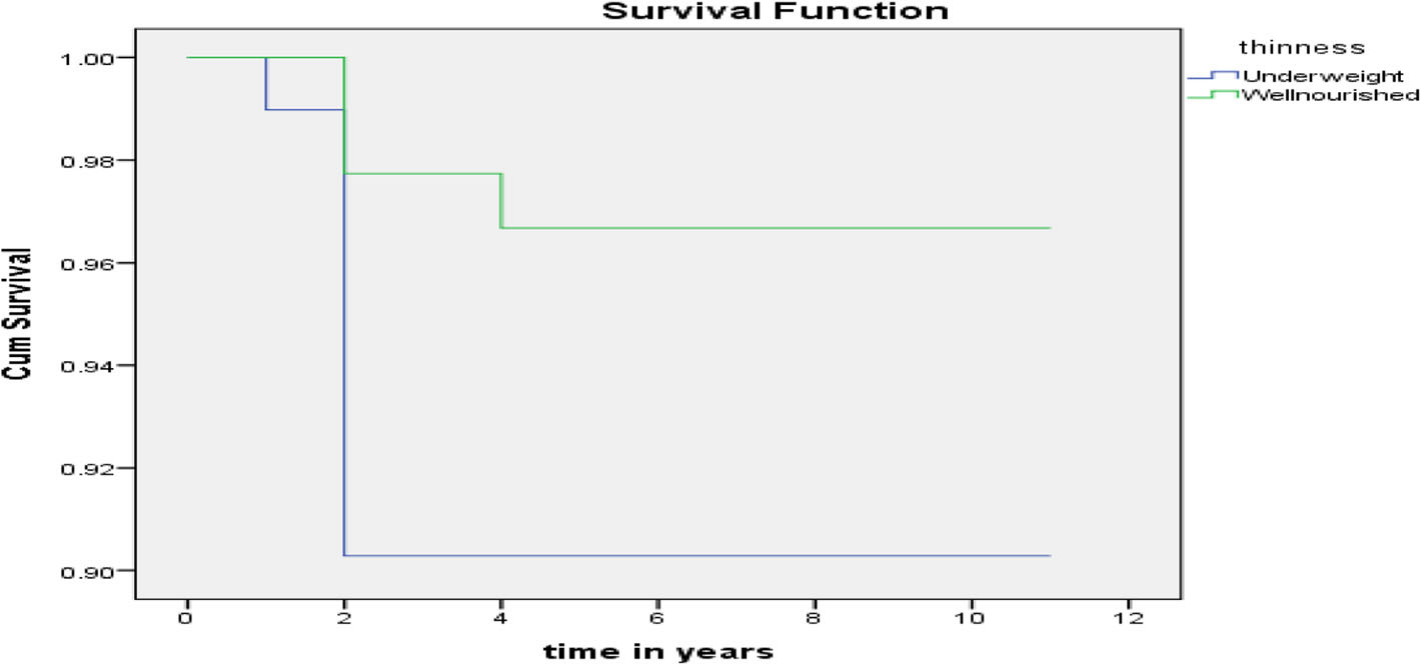
Survival function for children by their Nutritional status on ART follow up at Public Hospitals in Guraghe Zone, 2019 (Blue refers to survival lie for undernourished children while, green refers to survival graph for well nourished)

Majority of death among children were observed during the early period of ART start, in which 12 out of 13 deaths occurred during the first 2 years of ART start (Fig. 4). Even if the patter of death were similar, higher number of deaths (9 deaths) were observed among malnourished than well nourished (4 deaths) children.

Under Kaplan Meir test for comparison of mean survival time by nutritional status, there is significantly higher survival time among well nourished (11.1 years with 95% CI: 10.8 to 11.4) as compared to underweight children (9.76 with 95% CI: 9.19 to 10.32 years) (Table 5).

**Table 5:** Kaplan Meir test for comparison of mean survival time by nutritional status

	Median survival	se	Lower bound	Upper bound
Underweight	9.76	.289	9.19	10.32
Well nourished	11.10	.156	10.80	11.41
	χ^2^ (cox mantel)		4.59	
	P of log rank		0.032^a^	
	df (degree of freedom)		1	

^a^ Shows statistically significant difference in mean survival time of children

### Predictors of survival among ART children

Using both categorical and numerical covariates, cox proportional hazard regression model was done. The cox proportional hazard assumptions were fulfilled and checked using global test (*p* value of above 0.05). Younger children had shown to have higher hazards of death, which is per each year increase in age of child the hazards of death decrease by 14% (CHR = 0.86 95% CI: 0.75–0.99). Children from rural area had two fold increased hazards of death (CHR = 1.90; 95% CI: 0.58–6.12). Those children who were underweight (defines as low BMI for age z score below − 2 as compared to the WHO growth reference) shown to have almost four fold increased risk of early death as compared to well-nourished ones (CHR = 3.36; 95% CI: 1.03–10.9). Children with fair (CHR = 4.73; 95% CI: 1.4–14.9) and poor ART adherence (CHR = 4.73; 95% CI: 1.4–14.9) had more than four times increased hazards of death as compared to well adhered clients. In addition, those clients who were not supplemented with therapeutic foods had two times higher hazards of death (CHR = 2.18; 95% CI: 0.48–9.84) (Table 6).

**Table 6:** Cox regression showing Predictors of survival among ART children in Southwest Ethiopia, 2019

Variables	Options	Outcome		CHR with 95% CI	AHR with 95% CI	*P* value
		Death	Other			
Age	Per each year increase			0.86 (0.75–0.99)		
Sex	Male	10	106			
	Female	3	124	1		
Residence	Rural	9	125	1.90 (0.58–6.12)		
	Urban	4	105	1		
Nutritional status	Underweight	9	91	3.36 (1.03–10.9)	3.01 (0.80–11.4)	0.104
	Well nourished	4	139	1	1	
ART adherence	Good	7	195	1		
	Fair	5	29	4.73 (1.4–14.9)		
	Poor	1	6	4.03 (0.4–32.7)		
Therapeutic food	No	2	63	2.18 (0.48–9.84		
	Yes	11	167	1		
Eating disorder	No	1	79	1		
	Yes	12	151	2.44 (0.88–6.6)		
OIs	No	0.25	52	1		
	Yes	13	178	29.8 (0.11–78)		
ART start	Immediately	2	82	1	1	
	Later on	11	148	2.84 (0.63–12.82)	4.43 (0.84–23.34)	0.079
Baseline Hgb	Per each 1 g/dl increase in hgb			0.78 (0.63–.95)		
Anemia	Yes	7	203	2.38 (1.38–4.1)	1.55 (0.49–4.84)	0.453
	No	6	27	1	1	
WHO stage	WHO stage I	1	179	1	1	
	WHO stage II	7	32	35.2 (4.3–286)	25.1 (2.91–48.0)	0.003*
	WHO stage III	5	5	15.6 (1.8–34)	20.7 (2.16–38.3)	0.000*

* statistically significant at *p* value below 0.05 (5%)

Clients who started ART immediately with the same days of HIV confirmation had lower hazards of early death (CHR = 2.84; 95% CI: 0.63–12.82). Hazards of death was higher among children diagnosed with anemia as compared to non-anemic children (CHR = 2.38; 95% CI: 1.38–4.1). Similarly as the hemoglobin level rises, it showed 22% decline in hazards of death (CHR = 0.78; 95% CI: 0.63–.95). Significantly higher hazards of death was observed among advanced stage ART clients (WHO stage II and above) as compared to WHO stage I (Table 6).

After adjusting for confounder variables, multivariate cox proportional hazard regression was fitted. As a result, advanced WHO stage, nutritional status, duration of pre ART follow up and presence of anemia were significantly associated with increased hazards of death. Underweight children had almost three fold increase in hazard of death (AHR = 3.01; 95% CI: 0.80–11.4). Similarly children with anemia had higher hazards of death than children without anemia (AHR = 1.55; 95% CI: 0.49–4.84) (Table 6).

## Discussions

The findings from this study showed that a total of 13 (5.3%) children were dead during the course of their chronic HIV care with incidence density of 11.2 deaths per 1000 person years of follow up. Additionally, majority of deaths occurred during the first 2 years after the start of ART. Similarly review data on national level showed mortality rate of 5 to 8% at 6 month and 24 months of ART start [19]. Mortality rate (hazards of death was higher among males than females which is comparable to study in Iran which showed lower cumulative proportion surviving among males than in females *(P =* 0.0001) [20]. In addition study in Addis Ababa also showed about 8.8% mortality rate among children in which majority of death occurs during early periods of ART follow-up [21]. Study from Northern Ethiopia also showed mortality rate of about 8% [17].

In addition, nutritional status evidenced by being underweight and low hemoglobin had increased hazards of death. Underweight children had almost three fold increase in hazard of death (AHR = 3.01; 95% CI: 0.80–11.4). Similarly children with anemia had higher hazards of death than children without anemia (AHR = 1.55; 95% CI: 0.49–4.84). Similarly study among adult clients on HAART showed that being malnourished increased hazards of early death by 40% (AHR = 1.460, 95% CI (0.648, 3.287), *p* = 0.361] [18]. In addition pre ART nutritional status evidenced by low weight increased hazards of death (AHR = 5.4 95% CI 3.03–9.58) [22]. Also a study done in Ethiopia showed that, being malnourished (low weight) (AHR =4.99, 95% CI 2.4–10.2, *P* < 0.00) and low hemoglobin (HR = 2.92, 95% CI 1.3–6.7, *P* = 0.001) increase hazards of death among HIV clients respectively [17]. This emphasized that low weight and hemoglobin are usually indicative of advanced stage disease which decrease child survival significantly.

Thus, as the prevalence of malnutrition among children contributed to almost half of under-five mortality and affects up to 40% of children, maintaining good nutritional status of children is one of the primary proxy indicator for good treatment outcome [23]. This in turn will aggravate the bad cycles of HIV and malnutrition in that it will leads to advanced diseases and poor adherence to HIV chronic care [23, 24]. Significantly, higher number of HIV infected children had higher prevalence of underweight (77% versus 35%), stunting (65 and 61%) and wasting (63 and 26%) respectively [7].

Despite having nutritional rehabilitation, a fourfold increase in mortality is observed among HIV positives indicating the need for improved and focused nutritional care for children for improved treatment outcomes. In addition there should be high energy and micronutrient foods locally prepared in the community or some preparations like ready to use therapeutic foods or others should be readily available to the ART clients.

It estimated that about 3.2 million children living with HIV/AIDS, almost 90% cases were found in sub Saharan African countries in which malnutrition is common problem facing children [23]. This will interact with each other creating double burden among children in that malnutrition will reduce HAART effectiveness, response to drugs while increasing the risks of early death.

In addition, majority of children would not start ART before 6 months. Thus, children usually starts care later where children develop advanced stage disease including advanced WHO stage defining illnesses leading to lower survival [6]. Early initiation of ART usually improve the clinical outcomes of children. On the other side, in areas where ART coverage is about 54% [19, 25], late detection and initiation of treatment will deteriorate the nutritional status of children causing early mortality and immunological failure.

Despite its impressive methods and findings, as of any study the findings of this study should be seen in the light of some limitations. As the number of children on ART were smaller, the sample size was somewhat lower which might affect the power of the study. In addition, the secondary nature of the data makes, the availability of some factors like iron status and other nutritional indices unavailable.

## Conclusion and recommendations

Low nutritional status at the start of ART evidenced by underweight and anemia were found to be predictors of survival among HIV positive children. There should be improved, sustained and focused nutritional screening, care and treatment for children on ART follow up. In addition strong adherence support for chronic HIV care should be emphasized, so that it will be a significant input for improved nutritional status and ultimately improved treatment survival. The Health extension workers and the HIV support groups in collaboration with ART care givers should educate and promote care givers to enhance better nutritional support and care for children in addition to adherence to HAART therapy.

## Data Availability

All data generated or analyzed during this study are included in this published article.

## Abbreviations

A/CHR: Adjusted/Crude Hazard ratio
ART: Ante retroviral treatment
BMI: Body mass index
HIV/AIDS: Human Immune Virus/Acquired Immune deficiency syndrome
PLWHA: People living with HIV/AIDS
